# A Low-Stray-Inductance 1200 V/500 A SiC Power Module Based on Multilayer Insulated Metal Substrate

**DOI:** 10.3390/mi17050602

**Published:** 2026-05-14

**Authors:** Youyuan Yue, Liming Che, Cancan Li, Guangyin Lei

**Affiliations:** 1 Institute of Future Lighting, College of Intelligent Robotics and Advanced Manufacturing, Fudan University, Shanghai 200433, China; yyyue21@m.fudan.edu.cn (Y.Y.); 23110860045@m.fudan.edu.cn (L.C.); 22110860003@m.fudan.edu.cn (C.L.); 2 Research Institute of Fudan University in Ningbo, Ningbo 315336, China

**Keywords:** silicon carbide (SiC), insulated metal substrate (IMS), multilayer laminated substrate, high-thermal-conductive epoxy, stray inductance, mutual-inductance cancelling, transient thermal performance

## Abstract

With the growing need for high-power density, high-efficiency power electronics, wide band gap (WBG) semiconductors, such as silicon carbide (SiC) and gallium nitride (GaN), have been widely used in recent years. With high switching speed, stray inductance induced by packaging would cause voltage overshooting and oscillation during the switching transient, which should be mitigated at all costs. In this paper, a power module design based on a multilayer insulated metal substrate (MIMS) structure was proposed to effectively address the stray inductance concern based on the mutual-inductance cancelling effect. Fabrication process flow with high feasibility was also designed. Electrical and thermal simulations were conducted based on a power module with a nominal rating of 1200 V and 500 A. Compared to the planar module, the proposed design possessed much lower stray inductance (3.47 nH vs. 14.85 nH). In the transient thermal simulation, the proposed module exhibited a time constant 141.7% higher than that of the hybrid module with a ceramic substrate on the bottom but MIMS on the top, making it suitable for applications with high-constant power output requirements.

## 1. Introduction

Tremendous and continuous attention has been paid to silicon carbide (SiC) power semiconductors due to their higher switch speed, higher operating temperature, and higher thermal conductivity than that of Si devices [1,2]. To fully utilize the potential of SiC power devices, the stray inductance of the packaging needs to be mitigated due to its adverse effects of causing significant voltage spikes and current oscillation that may eventually lead to high switching loss or even the failure of the device [3,4,5].

To decrease the stray inductance of the package, different structures have been proposed, including the optimized wire-bonding structure, the planar structure with no bonding wires, and the hybrid structure. The first one suppresses the inductance through the modulation of the current commutation loop. Chen et al. put the switch device close to its freewheeling diode instead of its antiparallel diode to shorten the length of the commutation loop, and the resultant loop inductance was about 35% lower than the conventional design [6]. Such a design, however, possesses limited improvement due to the remaining bonding wires and the flattened current path. Furthermore, the planar designs that substitute conductive components, such as Cu units [7,8], molybdenum (Mo) buffer layers [9,10], soldering balls [11,12], or PCBs [13,14] for bonding wires, and introduce 3-D current paths have been widely studied. Horio et al. replaced bonding wires with Cu pins and designed laminated inductor layers. Due to the shortened current path and the mutual-inductance cancelling effect, the inductance decreased from nearly 50 nH for the traditional structure with Al bonding wires to 22.3 nH [8]. This structure did not optimize the design of power terminals; the total inductance, therefore, was still relatively high for SiC power modules. Knoll et al. embedded SiC dies into PCB and completed electrical connectivity through Cu-filled microvias [13]. Thanks to the integration advantages of PCB, a low inductance of 2.3 nH in the power loop was achieved. Due to the poor thermal conductivity of PCB, however, such a design requires careful consideration in the tradeoff between the thermal and electrical performance of the module, which significantly increases the design difficulty. Sun et al. proposed a substrate-embedded SiC module and replaced the bonding wires with fan-out interconnection layers [7]. A very low power loop inductance of 155 pH was achieved, but at the cost of a quite complex fabrication process and poor thermal management. The third solution—the hybrid structure or planar packaging with bonding wires—retains the conventional connection process and utilizes the mutual-inductance cancelling effect, thus combining the benefits of the two schemes discussed above, i.e., ease of fabrication and a marked reduction in inductance [15,16,17]. Tanimoto et al. designed a multilayer direct-bonded copper (DBC) substrate, including three Cu layers and two ceramic layers to allow the forth and back current flow in the first and second Cu layers, respectively, which strongly enhanced mutual-inductance cancelling and limited the loop inductance to about 4.5 nH [16]. However, the fabrication of ceramic to achieve electrical connectivity between different layers is relatively troublesome, and the multilayer DBC substrate may increase the heat resistance, which hinders the practical application of such designs.

Though various measures have been proposed to reduce the stray inductance of the module, developing multilayer substrates manifests remarkable effectiveness. In this paper, to overcome the producibility and thermal-dissipating concerns of the traditional multilayer ceramic substrates, high-thermal-conductive epoxy (HTCEP) was chosen as an insulator, and a layer-by-layer lamination process flow was developed to fabricate multilayer IMS (MIMS). Based on this, a fabrication process of a 1200 V/500 A SiC power module was also proposed, which was compatible with the lamination flow of the MIMS. The stray inductance was extracted, and the steady and transient thermal simulations were conducted for the proposed module. Simulation results were then compared with the traditional planar module based on a ceramic substrate and a hybrid module with a ceramic substrate on the bottom and MIMS on the top. The results validated the much lower stray inductance and better thermal performance of the proposed module.

## 2. Module Design

### 2.1. Module Requirements Analysis

To achieve the 1200 V voltage level, Sichain’s (Ningbo, China) 1200 V/127 A SiC MOSFET die (SG2M016120B) was selected. Considering the current capability of 500 A of the module, 4 dies were needed in parallel for each switch.

### 2.2. Module Structure Design

**Figure 1:**
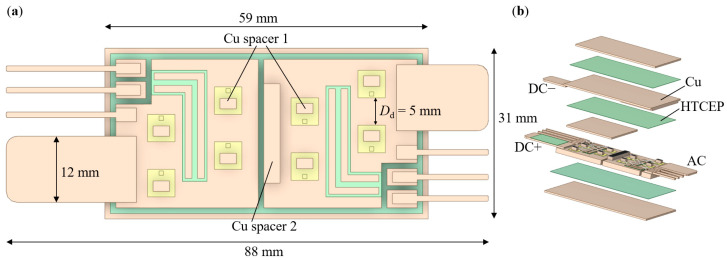
Structure of the IMS module: (**a**) bottom IMS layout; (**b**) exploded view.

**Figure 2:**
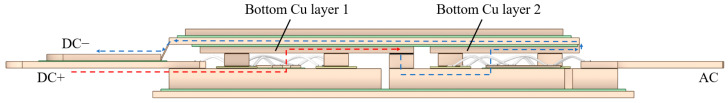
Schematic of the current commutation loop in the IMS module.

Based on the 4-die parallel configuration, a low-stray-inductance SiC module package structure with MIMS was proposed, as shown in Figure 1. For each switch, i.e., high side and low side switch (HS and LS), 4 dies were equally divided into two rows placed perpendicular to the direction of the main current flow. The achievement of the low stray inductance was mainly attributed to the following points: MIMS on the top side of the module, the laminated DC+ and DC− terminals, and the replacement of Al bonding wires with Cu spacers. HTCEP was utilized to form the planar structure in the power loop. Figure 2 illustrates the current commutation loop of the module. Current flowed into the module through the DC+ terminal, then passed through the MOSFETs of the HS, Cu spacers 1, the bottom Cu layer 1 of MIMS, then the Cu spacer 2, MOSFETs of the LS, Cu spacers 1, the bottom Cu layer 2 of MIMS, and the middle Cu layer of MIMS, finally flowed out of the module from the DC− terminal. From this commutation loop, it was obvious that the current flowed through the bottom and middle Cu layers of the MIMS, and the laminated DC+/− terminals were in the opposite direction. Due to the mutual-inductance cancelling effect, the total inductance of the loop can be significantly decreased [3,8]. Moreover, owing to the high flexibility of the MIMS, the Cu layer 2, the middle Cu layer, and the DC terminal were located in the same Cu component, which eliminated the bonding wires or the metal-filled vias to conduct between different Cu layers and reduced one connecting step for the terminal; thus, it further decreased the inductance and simplified the fabrication flow. Additionally, compared to the bonding wires or thin Cu clips, the Cu spacers with a larger cross-sectional area also helped to reduce the power loop inductance [3].

For the gate signal loop, thin Cu strips with a thickness of 0.3 mm were substituted for traditional Cu trace etched on the top layer of the substrate. Such a design preserves the geometry integrity of the top Cu layer of the bottom IMS and benefits the thermal performance of the module. For the signal loop, to simplify the manufacturing process of the module, 5 mil Al bonding wires were soldered between the gate/source pads of the dies and the Cu strips to complete the signal transferring loop.

Owing to the high thermal expansion match of the epoxy with Cu compared to the ceramic (Al_2_O_3_, AlN, Si_3_N_4_, etc.) used in the DBC or the active metal brazing (AMB) substrates [18], the thickness of the Cu layers attached to the two sides of the IMS can be different. Previous research has confirmed that with a thicker top metal layer, the thermal performance of the IMS increased [19]. However, the thickness of the former was usually below 2 mm [20,21,22,23]. Herein, to fulfill the potential of the IMS, we chose a thick Cu bulk with a thickness of 2.5 mm as the top layer of the bottom IMS. A thicker Cu layer exhibits higher thermal capacity, which contributes to the decrease in the junction temperature (*T*_j_) changes of the MOSFET during normal operation. While the bottom Cu layer of the bottom IMS provides mechanical support to the module and allows ease of sintering/soldering the attachment to the heatsink. This Cu sheet, therefore, can be relatively thin, and a thickness of 0.8 mm was chosen in this module.

An important geometry parameter in the substrate layout design is the distance between two adjacent dies (*D*_d_). On the one hand, increasing *D*_d_ results in dies far from each other, which reduces the thermal coupling effect between the dies and benefits the thermal performance of the module [24,25]. Large *D*_d_, on the other hand, enlarges the Cu layer on the IMS, thus limiting the power density of the module. In the proposed module structure, thanks to the high thermal dissipation capability of the IMS, *D*_d_ was determined to be a relatively small value of 5 mm, which controlled the footprint of the IMS and stray inductance without significantly worsening the thermal performance of the module.

**Figure 3:**
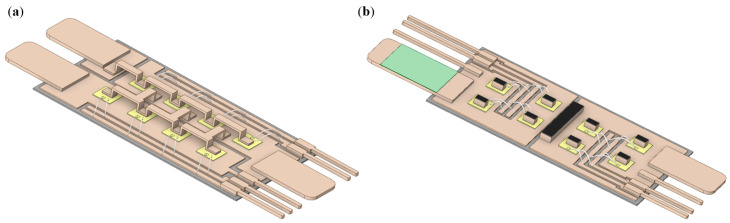
Structure of (**a**) AMB module and (**b**) hybrid module (with top MIMS hidden).

Moreover, for comparison, a traditional single-sided cooling module packaged with AMB substrates and a hybrid double-sided cooling module packaged with an AMB substrate on the bottom and MIMS on the top were also designed. The structures of the two modules are shown in Figure 3. The AMB substrate contains 3 layers (Cu/Si_3_N_4_/Cu), with thicknesses of 0.3/0.32/0.3 mm, respectively, which is a common choice and also satisfies the insulating requirement of the modules [26,27,28]. The *D*_d_ value of the two modules was 5 mm—the same as the proposed IMS module. Specifically, due to the small area of the source pads and high current level of the dies, Cu clips instead of Al bonding wires were chosen to achieve the connection of the power loop in the AMB module.

The dimensions and power densities of the three modules without terminals and before encapsulation are summarized in Table 1. The footprints of the modules were similar, but with different thicknesses, the final volumes and power densities varied. Notably, though the IMS module exhibited the lowest power density, it adopted a baseplate-less design, which means that the heatsink can attach to the Cu layer of the IMS directly without inserting a baseplate. This could not only further improve the heat-dissipating performance but also decrease the volume of the cooling system.

## 3. Properties Simulation of the Module

Finite-element simulations were conducted on the electrical and thermal properties of the proposed module to confirm the effectiveness of the design. The main physical and geometrical properties of the materials in the module used for the simulation are listed in Table 2.

### 3.1. Extraction of the Stray Inductance

**Figure 4:**
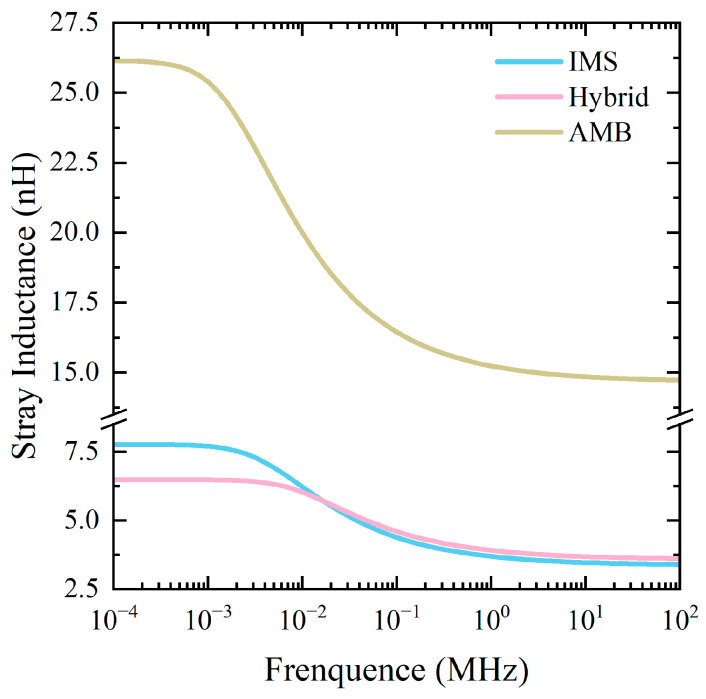
Stray inductance of the IMS, hybrid, and AMB modules with changing frequency.

Stray inductance values of the power loop were extracted by Ansys Q3D. As seen in Figure 4, the stray inductance of the three modules decreased when increasing the frequency. At 10 MHz, the stray inductance of the proposed IMS module and hybrid module were quite close (3.47 and 3.69 nH, respectively). For the AMB module, the stray inductance was 14.85 nH. Compared to the commercial 1200 V/660 A SiC power module DCM™ 1000X (6.5 nH) [29], the proposed IMS module achieved a 46.7% decrease in stray inductance. The significantly lower stray inductance of the IMS and hybrid modules than that of the AMB and DCM™ 1000X modules demonstrated the effectiveness of the mutual-inductance cancelling effect and the replacement of the spacers with bonding wires or thin clips in decreasing the total inductance.

### 3.2. Simulation of the Steady Thermal Properties

**Figure 5:**
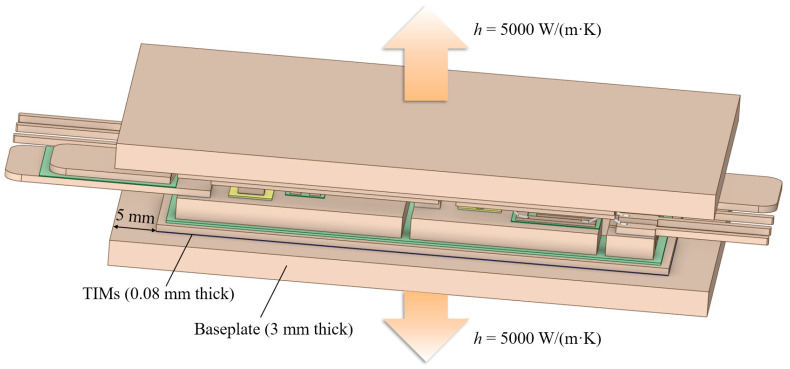
Setup of the modules (IMS module as an example) in the thermal simulations.

For thermal simulations, the power losses *P* of the die were needed. Since this article mainly focused on the comparison between the different modules, 100 W was assigned to each die, and the heat source region was set to be the whole die to simplify the simulation process. As shown in Figure 5, the modules were placed on the Cu baseplate (3 mm thick) with a layer of thermal interface materials (TIMs, silicone grease in this paper). Then, the thermal transferring coefficient *h* of the bottom and top (for the IMS and hybrid modules) surfaces of the baseplates was set to be 5000 W/(m^2^·K). The ambient temperature *T*_a_ was assumed to be 25 °C. These boundary conditions are summarized in Table 3. Some necessary simplification, such as the removal of the gate pads and the bonding wires, was performed to save the computing resources in both the steady and transient thermal simulations. In both thermal simulations, the modules were under hexa-unstructured meshing and shared similar settings of the max element size.

In particular, to investigate the effect of HTCEP on the thermal performance of the module and simplify the transient thermal simulation, six types of HTCEP with a series of equal-difference thermal conductivities, i.e., 2.5, 5, 7.5, 10, 12.5, and 15 W/(m·K), respectively, were applied as the insulator. The corresponding IMS and hybrid modules were named IMS-2.5 to IMS-15 and hybrid-2.5 to hybrid-15, respectively.

**Figure 6:**
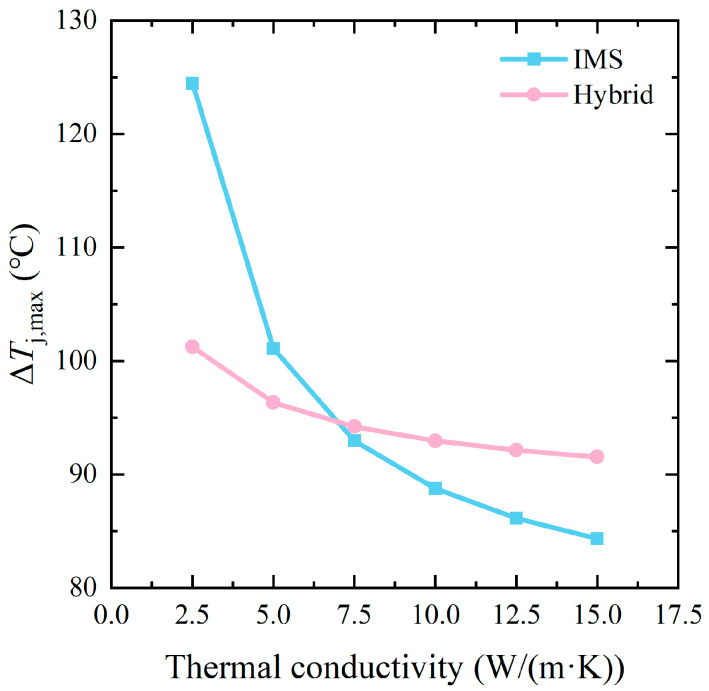
*T*_j,max_ of the IMS and hybrid modules with HTCEP with different thermal conductivity.

Simulations were conducted with Ansys Icepak. The results of the steady thermal performance of the IMS and hybrid modules with different HTCEP are shown in Figure 6. While the maximum junction temperature change (Δ*T*_j,max_) of the AMB module was 158.37 °C, this much higher value mainly resulted from its single-sided cooling design. As seen in Figure 6, the Δ*T*_j,max_ of both the IMS and hybrid modules decreased with increasing thermal conductivity of the HTCEP. This was because the HTCEP with high thermal conductivity decreased the thermal resistance of the IMS and enhanced the heat conduction from the dies to the coolant. The maximum junction temperature decreased monotonically when increasing the thermal conductivity of the HTCEP and then became less sensitive when further increasing the thermal conductivity. For IMS modules, the critical point was about 10 W/(m·K). Considering the heat-transferring process in the module, the heat generated by the dies was mainly conducted to the outward surface of the baseplates through the different layers and then dissipated to the coolant by convection. With the increasing thermal conductivity of the HTCEP, the dominant barrier of the heat-transferring process changed from the conduction in the substrates and the baseplates to the heat convection between the baseplates and the coolant, i.e., the heat resistance of the substrates became less important. Therefore, the decreasing rate of Δ*T*_j,max_ was high at first and then declined. Notably, the decrease in Δ*T*_j,max_ of the IMS module was much more pronounced than that of the hybrid module. Due to the higher heat resistance of the upward heat transferring path compared to the downward one, the latter became the main heat-dissipating path of the module. The bottom substrate of the hybrid module was the AMB substrate, which did not change with different HTCEP. Therefore, the main heat-dissipating path of the hybrid modules was not affected by the increasing thermal conductivity of the HTCEP, and Δ*T*_j,max_ exhibited a smaller reduction than that of the IMS modules.

**Figure 7:**
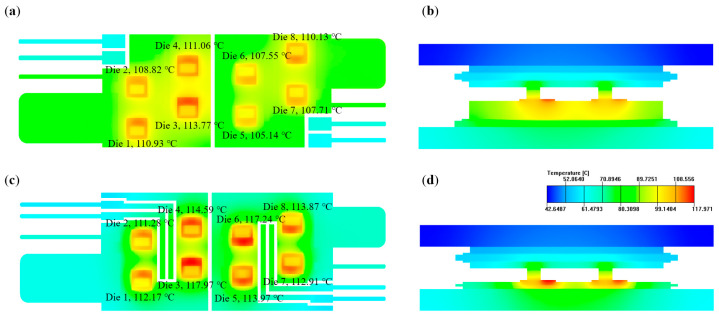
Temperature distribution of the (**a**) substrate and (**b**) cut plane of IMS-10 module; and (**c**) substrate and (**d**) cut plane of the hybrid-10 module.

Moreover, the Δ*T*_j,max_ values of the two modules were quite close under the thermal conductivity of 7.5 W/(m·K). When further increasing the thermal conductivity, the Δ*T*_j,max_ of the IMS module was much lower than that of the hybrid module. According to:

(1) $$R_{th}=\frac{L}{kA},$$

where *R*_th_ (K/W) is the thermal resistance; *L* (m) is the length of the materials parallel to the thermal transferring direction, *k* (W/(m·K)) is the thermal conductivity of the materials, and *A* (m^2^) is the cross-section area of the materials perpendicular to the thermal transferring direction; the thermal conductivity of the HTCEP (2.5 to 15 W/(m·K)) was significantly lower than that of the Si_3_N_4_ (90 W/(m·K)), while the thickness of the HTCEP layer was close to that of the Si_3_N_4_ layer (0.27 to 0.32 mm), therefore, the thermal resistance of the former was much higher. In Figure 6, however, with the increasing thermal conductivity, the IMS modules exhibit better steady-state thermal performances than the hybrid modules. This was mainly attributed to the thick and undivided Cu bulks on the topside of the IMS. Figure 7 shows the temperature distribution of the IMS-10 and hybrid-10 module. From Figure 7a,c, it can be seen that the etched traces on the AMB substrate hindered the heat spread on the substrate surface, while the IMS did not show this weakness. In Figure 7b,d, it is evident that the thick Cu bulk on the IMS effectively enhanced the heat dispersion to the surroundings due to the much longer vertical length than the thin Cu layer on the AMB substrate. In other words, the Cu bulk on the IMS facilitated the heat spread in all three directions, which endowed the IMS module with a better steady-state thermal performance than the hybrid module.

Figure 7 also provides the *T*_j_ of the dies in the modules. *T*_j,max_ located in die 3 in both the modules. The high *T*_j_ of die 3 mainly resulted from the very close distance to the edge of the Cu bulk. Thermal coupling with the adjacent dies 1, 2, and 4 further increased the *T*_j_ of die 3, making it the highest value in eight dies.

### 3.3. Simulation of the Transient Thermal Properties

Another superiority of the IMS module lies in the transient thermal condition. To balance the properties and material costs, IMS-10 and hybrid-10 modules were chosen as examples to conduct the transient thermal simulation. Power losses *P* were changed to 50 W/die, while the thermal transferring coefficient *h* was still 5000 W/(m^2^·K), as mentioned above. Since *T*_j,max_ occurred in die 3, its *T*_j_ was chosen in the next transient thermal simulation.

**Figure 8:**
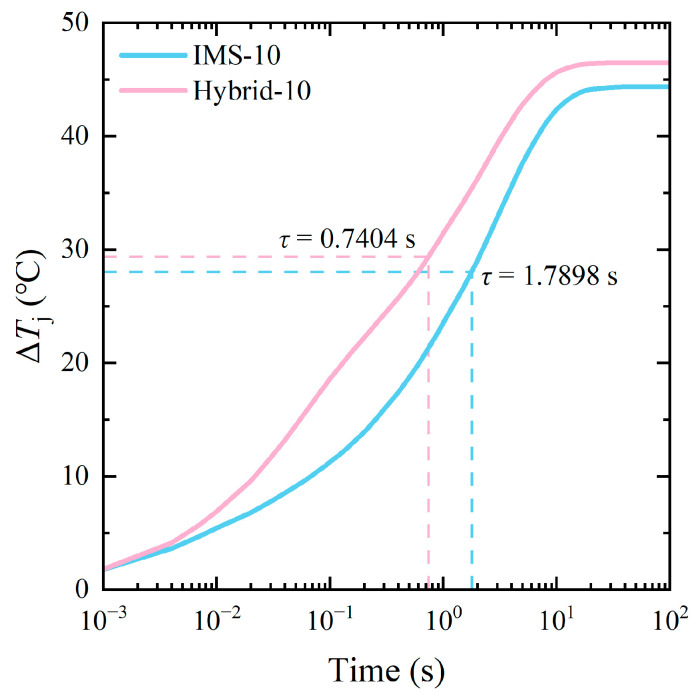
*T*_j_ increasing curves of the IMS-10 and hybrid-10 modules in 100 s.

Figure 8 presents the increasing *T*_j_ of the two modules over 100 s. With the dies continuously working, the *T*_j_ also steadily increased and finally became steady. Obviously, the increasing rate of *T*_j_ of the IMS-10 module was lower than that of the hybrid-10 module. The final steady value of *T*_j_ of the former was also lower, which was consistent with the results of the steady-state thermal simulation discussed above. Time constant *τ* was used to describe how fast the system responded to the external change. During the heating-up circumstance, the value of *τ* typically equals the time that the system takes to reach (1 − 1/e) times the final steady temperature. As presented in Figure 8, *τ* of the IMS-10 module was 1.7898 s, which was 141.7% higher than that of the hybrid-10 module. Lower Δ*T*_j_ and higher *τ* weaken the thermal shock in the dies and the nearby sintering layers, suppress the development of cracks, and finally enhance the reliability of the module [30,31].

The reason for the slower change rate of *T*_j_ and the lower Δ*T*_j_ of the IMS module mainly lies in the thick Cu bulk in the bottom IMS. With the high thermal capacity exhibited by the Cu bulk, its temperature changes slowly when absorbing the heat generated from the dies [32]. This heat-pool-like behavior effectively adjusts the heat transportation of the dies and finally contributes to the tardier *T*_j_ changing and lower Δ*T*_j_ of the IMS module.

## 4. Fabrication of the Module

### 4.1. Lamination of the IMS and MIMS

**Figure 9:**
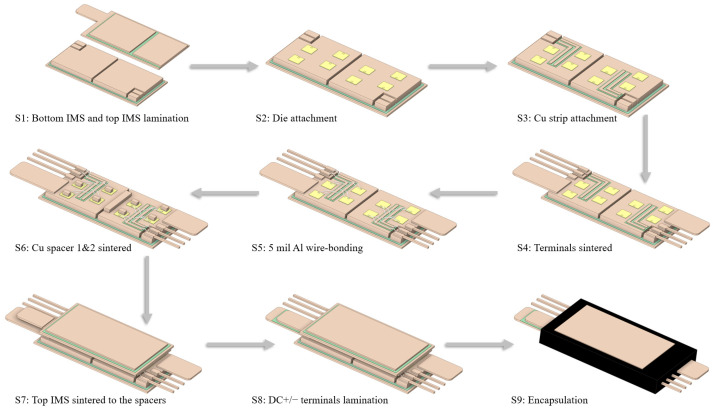
Steps of the fabrication process of the IMS module.

In this section, the fabrication flow of the proposed module is illustrated. As shown in Figure 9, the whole fabrication process includes 9 steps. The first step was the lamination of the IMS. Owing to the good adhesive capacity of the HTCEP, the MIMS can be easily fabricated through a layer-by-layer stacking process. An HTCEP layer (with a thickness of 0.27 mm), which serves as an insulator between Cu layers, was cut into a specific geometry, removed from the release film on one side, and pre-sticked to the bottom Cu layer. The combination was then heated by the pre-stick machine and cooled down to room temperature according to the instructions. After that, the release film on the other side of the HTCEP was removed, and another Cu layer was stacked upon the HTCEP.

For the MIMS on the topside of the module, the stacking process followed similar steps as those mentioned above, but with more layers. Particularly, the Cu sheet connected to the LS was then folded up clockwise at an angle of 180° to cover the HTCEP layer and served as the middle conductor layer of the MIMS.

To form reliable bonding between the HTCEP and Cu, a hot-press was necessary to cure the HTCEP according to the instructions. Afterwards, the whole lamination process of the IMS was completed.

### 4.2. Assembly of the Module

After completing the fabrication of IMS, dies were attached to the top Cu layer of the bottom IMS (S2). Notably, the whole fabrication flow of the module comprises several connection processes. To simplify the fabrication process, Ag sintering was adopted as the interconnection material instead of reflow soldering since the latter required the use of several types of soldering materials with different melting temperatures to ensure that the already completed soldering layer did not melt during the following soldering process [17]. For S3, Cu strips were attached to the top Cu layer of the bottom IMS with HTCEP layers (with a thickness of 0.09 mm), which were hot-pressed and cured. Next, in S4, the terminal frame was sintered to the bottom IMS, and the auxiliary connection parts were then removed. For S5, connections for the gate and source pads of the dies, Cu strips, and signal terminals were completed through 5 mil Al bonding wires to form the gate connection.

In the subsequent S6, Cu spacers 1 and 2 were sintered on the source pad of the dies and the bottom IMS, respectively. Specifically, since spacer 1 was placed on the dies with a certain thickness, the sintering paste under spacer 2 was a little thicker than that of spacer 1 to ensure both types of components were sintered simultaneously. Then, in S7, the MIMS was sintered with the Cu spacers. Next, in S8, an HTCEP layer (with a thickness of 0.09 mm) was placed between the DC+/− terminals, and these three components were then hot-pressed to achieve the laminated terminals.

Finally, for S9, the whole module was encapsulated with an epoxy molding compound (EMC) for insulation and protection. Here, the EMC should have the matched CTE with the IMS to mold around the dies rigidly, thus relieving the thermal stress in the sintering layer under the dies [33]. To adopt the baseplate-less design, the outward Cu layers on the top and bottom of the module protruded from the EMC slightly.

## 5. Conclusions

In this paper, a MIMS structure and a 1200 V/500 A double-sided-cooling SiC power module with low stray inductance based on the MIMS was proposed. A layer-by-layer lamination process was introduced to fabricate the MIMS and then the proposed module. Stray inductance extraction and thermal simulations were conducted to verify the properties of the proposed module. For comparison, a traditional single-sided-cooling AMB module and a hybrid module with an AMB substrate on the bottom and MIMS on the top were also designed with in-depth simulations. Thanks to the mutual-inductance cancelling effect that occurred in the MIMS and the laminated DC+/− terminals, and the alternation of the Cu spacers to the Cu clips, the proposed IMS module exhibited markedly lower stray inductance than the AMB module (3.47 to 14.85 nH). Under steady thermal conditions, the *T*_j,max_ of the proposed IMS module decreased with the increasing thermal conductivity of the HTCEP (from 2.5 to 15 W/(m·K)). With an HTCEP of 10 W/(m·K), the Δ*T*_j,max_ of the IMS module decreased by 44% and 4.5%, respectively, compared to the AMB and hybrid modules. When further increasing the thermal conductivity of the HTCEP to 15 W/(m·K), the former presented a Δ*T*_j,max_ decrease of 7.9% compared to the hybrid module. This was mainly attributed to the double-sided cooling and thick Cu bulks, which enhanced the thermal spreading in the three dimensions. Under the transient condition, the proposed IMS module showed a much slower increase in *T*_j_, with a time constant *τ* 141.7% higher than that of the hybrid module. Such superiority was mainly attributed to the high thermal capacity exhibited by the thick Cu bulk in the IMS module, allowing it a promising design to develop high-performance miniaturized SiC power modules.

## Figures and Tables

**Table 1:** Dimensions and power densities of the modules.

	Footprint (mm × mm)	Thickness (mm)	Volume (mm^3^)	Power Density (kW/cm^3^)
IMS	59 × 31	8.43	15,418.5	38.9
Hybrid		5.78	10,571.6	56.8
AMB		3.76	6877.0	87.2

**Table 2:** Physical and geometrical properties of the materials used in the module.

	Thickness (mm)	Density (kg/m^3^)	Specific Heat (J/(kg·K))	Thermal Conductivity (W/(m·K))
Cu	varies	8933	381	395
HTCEP	0.09, 0.27	1675	1150	2.5 to 15
Si_3_N_4_	0.32	3750	896	90
SiC	0.18	3210	677.8	340
Sintered Ag	0.08	10,524	236	200
TIMs	0.08	3200	234	3

**Table 3:** Boundary conditions of the thermal simulations.

Parameter	Value
Power loss *P* (W)	100, 50
Ambient temperature *T*_a_ (°C)	25
Thermal transferring coefficient *h* (W/(m^2^·K))	5000

## Data Availability

The original contributions presented in this study are included in the article. Further inquiries can be directed to the corresponding author.
